# Supplementary material for: Effects of occlusal disharmony on cardiac fibrosis, myocyte apoptosis and myocyte oxidative DNA damage in mice
Source: PLoS One. 2020 Jul 27;15(7):e0236547. doi: 10.1371/journal.pone.0236547 (PMC7384634; doi:10.1371/journal.pone.0236547)
Supplement: S1 Data — (PDF) [file pone.0236547.s001.pdf]

Supplementary Figure 1

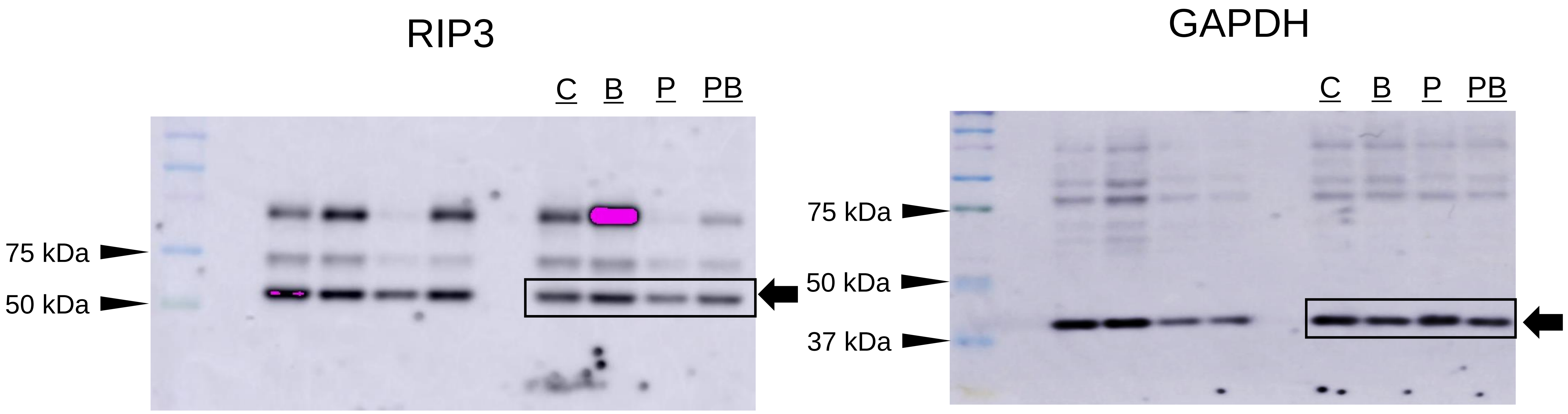

Supplementary Figure 1. Representative full-length immunoblots of Fig. 4A. The amount of RIP3 and GAPDH was shown. The black-line box indicated by arrow in each blot is corresponded to the cropped parts that are showed in the main article. C: control, B: BO, P: Pro, PB: Propranolol + BO.

Supplementary Figure 2

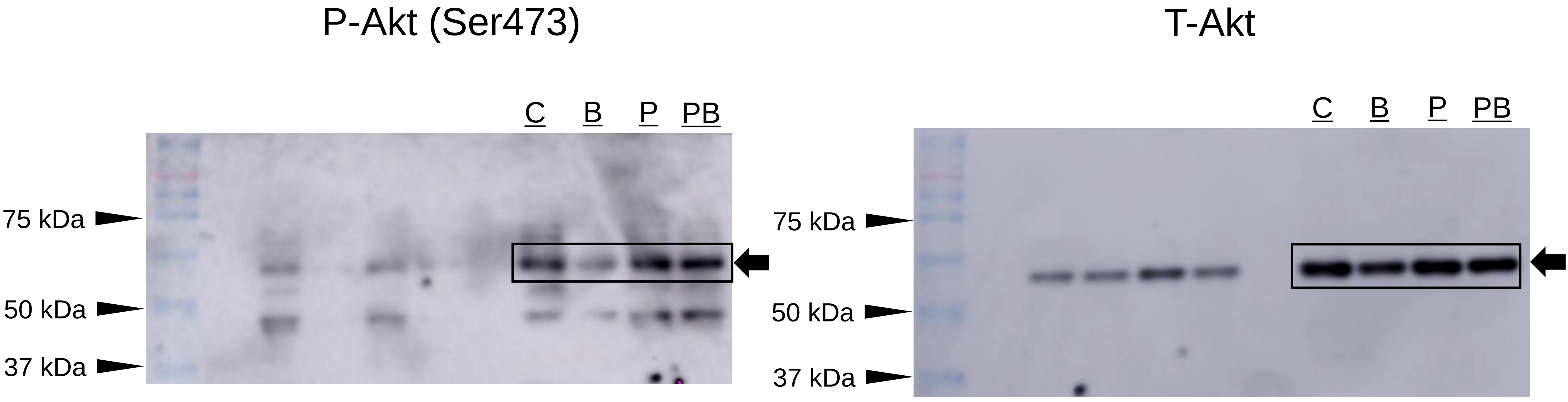

Supplementary Figure 2. Representative full-length immunoblots of Fig. 4B. The amount of phosphorylated Akt (Ser 473) and total Akt was shown. The black-line box indicated by arrow in each blot is corresponded to the cropped parts that are showed in the main article. C: control, B: BO, P: Pro, PB: Propranolol + BO.

Supplementary Figure 3

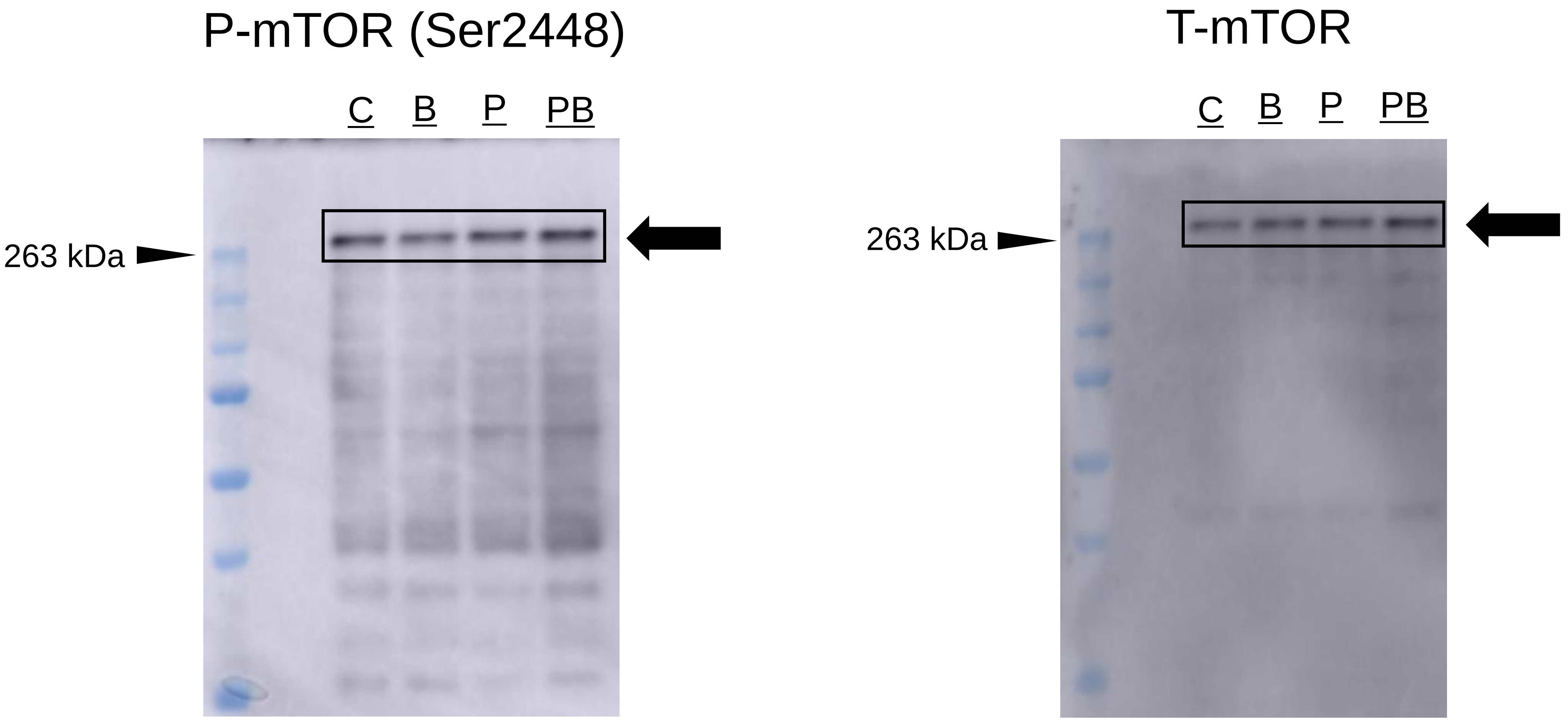

Supplementary Figure 3. Representative full-length immunoblots of Fig. 4C. The amount of phosphorylated mTOR (Ser 2448) and total mTOR was shown. The black-line box indicated by arrow in each blot is corresponded to the cropped parts that are showed in the main article. C: control, B: BO, P: Pro, PB: Propranolol + BO.

Supplementary Figure 4

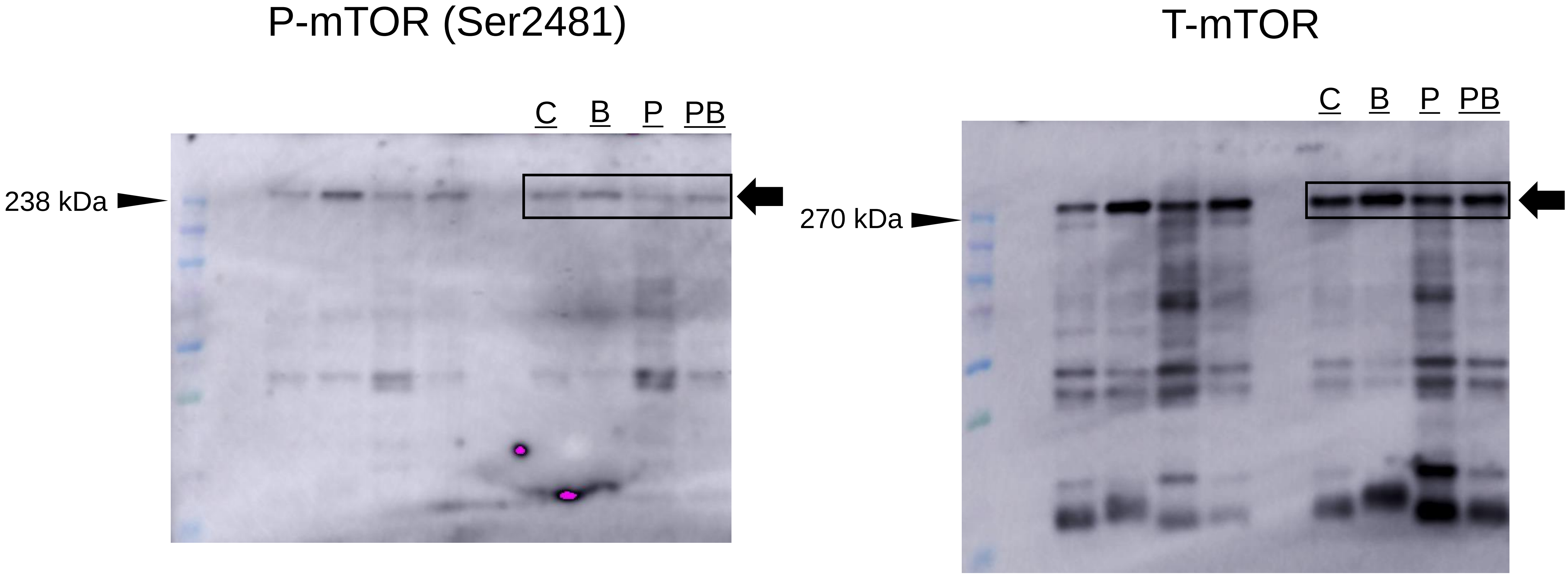

Supplementary Figure 4. Representative full-length immunoblots of Fig. 5A. The amount of phosphorylated mTOR (Ser 2481) and total mTOR was shown. The black-line box indicated by arrow in each blot is corresponded to the cropped parts that are showed in the main article. C: control, B: BO, P: Pro, PB: Propranolol + BO.

Supplementary Figure 5

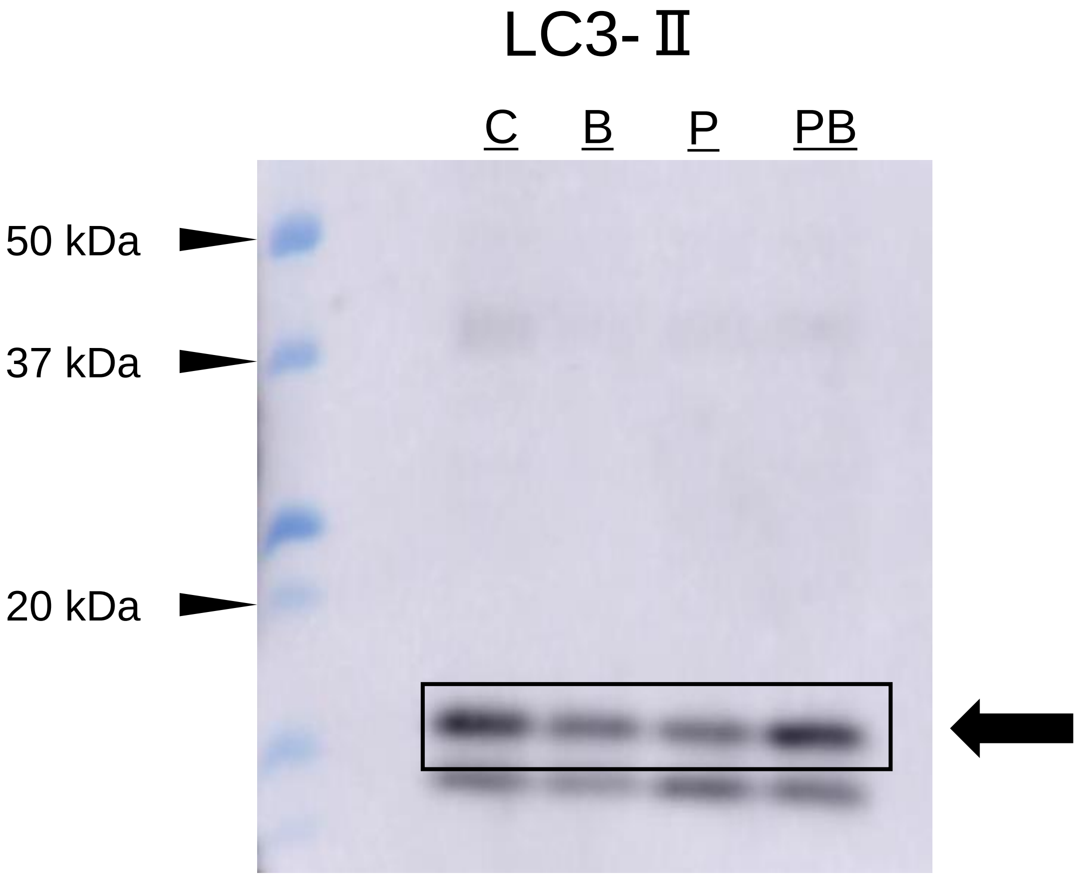

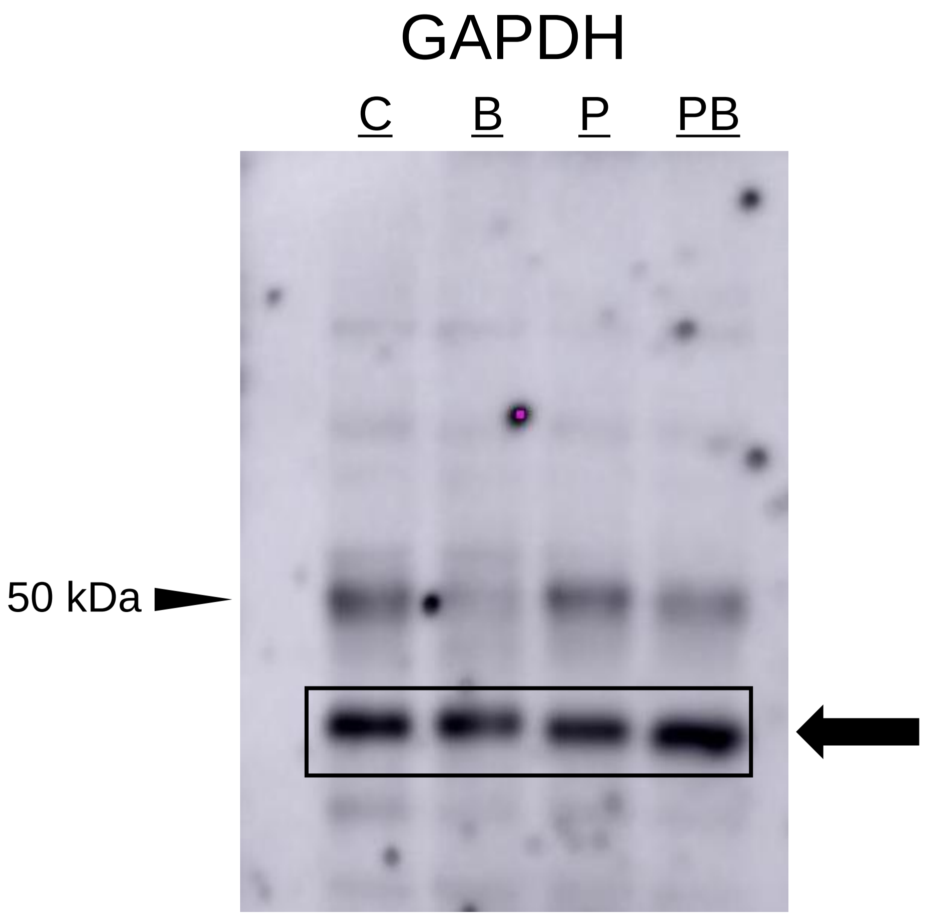

Supplementary Figure 5. Representative full-length immunoblots of Fig. 5B. The amount of LC3-Ⅱ at and GAPDH was shown. The black-line box indicated by arrow in each blot is corresponded to the cropped parts that are showed in the main article. C: control, B: BO, P: Pro, PB: Propranolol + BO.

Supplementary Figure 6

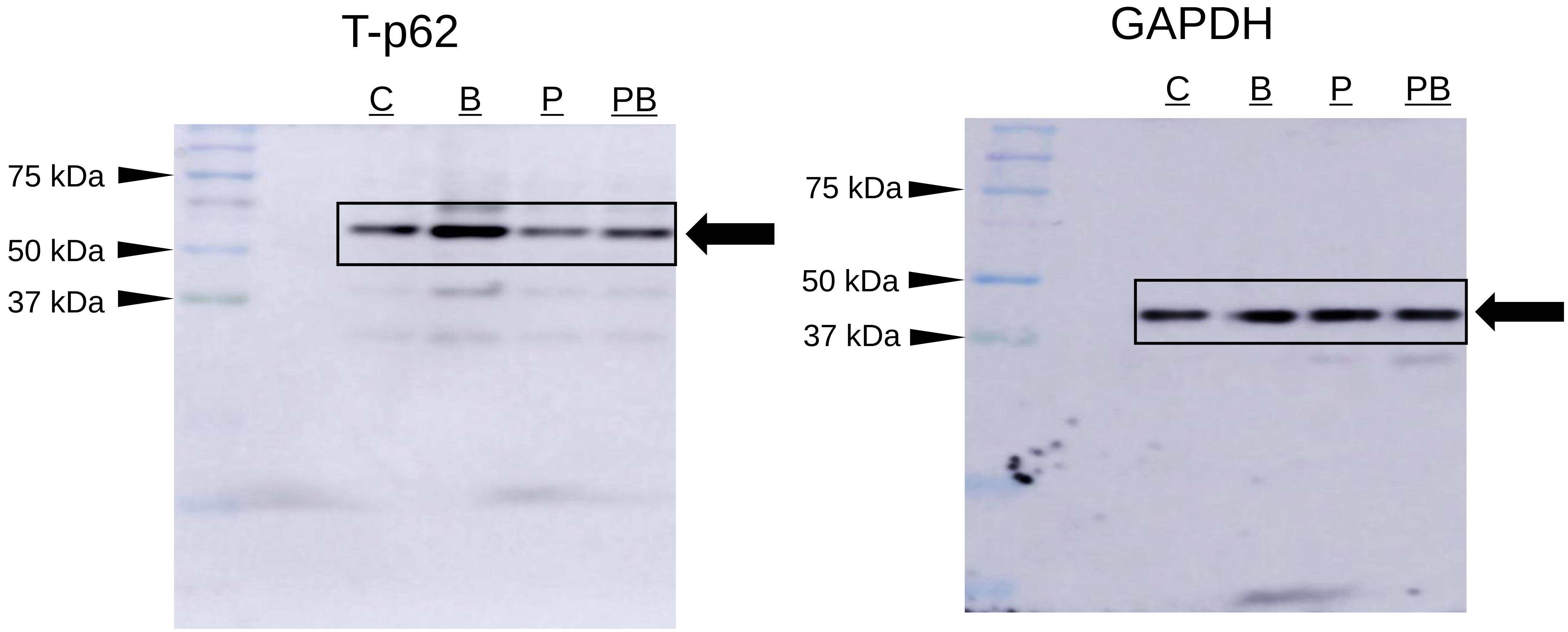

Supplementary Figure 6. Representative full-length immunoblots of Fig. 5C. The amount of T-p62 at and GAPDH was shown. The black-line box indicated by arrow in each blot is corresponded to the cropped parts that are showed in the main article. C: control, B: BO, P: Pro, PB: Propranolol + BO.
