# Supplementary material for: Effects of occlusal disharmony on cardiac fibrosis, myocyte apoptosis and myocyte oxidative DNA damage in mice
Source: PLoS One. 2020 Jul 27;15(7):e0236547. doi: 10.1371/journal.pone.0236547 (PMC7384634; doi:10.1371/journal.pone.0236547)
Supplement: S2 Data — (PDF) [file pone.0236547.s002.pdf]

Fig S1

		P value (Bonferroni)
Control N=6	BO	1.6×10^{-27}
	Pro	1.0
	BO＋Pro	2.4×10^{-27}
BO N=8	Control	1.6×10^{-27}
	Pro	8.2×10^{-28}
	BO＋Pro	1.0
Pro N=6	Control	1.0
	BO	8.2×10^{-28}
	BO＋Pro	1.2×10^{-27}
BO＋Pro N=10	Control	2.4×10^{-27}
	BO	1.0
	Pro	1.2×10^{-27}

Treatment effects : $P = 1.81049 \times 10^{-38}$, total sample size = 8
Time effects : $P = 4.9 \times 10^{-16}$, total sample size = 8
Two way ANOVA with repeated measures followed by Bonferroni *post hoc* test

Fig S1.
Statistical analysis of Fig 1B.

Fig S2

A

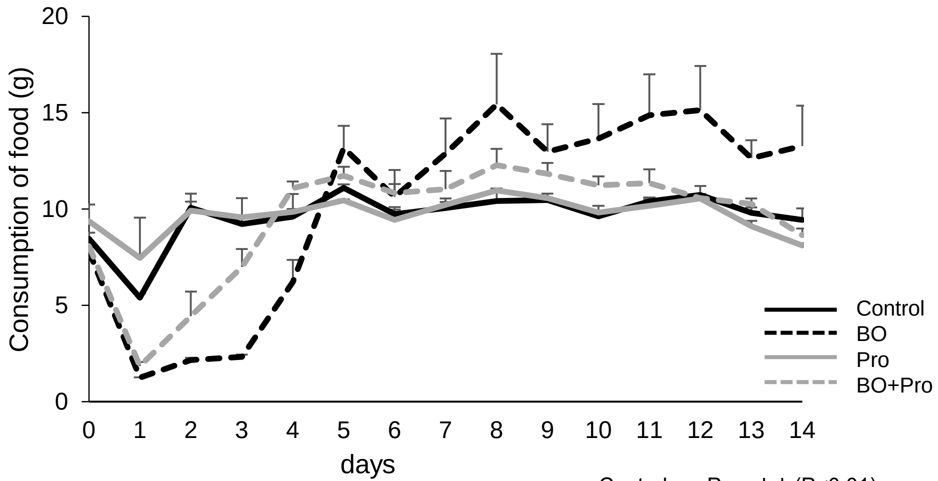

B

		P value (Bonferroni)
Control N=6	BO	1.0
	Pro	1.0
	BO＋Pro	1.0
BO N=8	Control	1.0
	Pro	1.0
	BO＋Pro	1.0
Pro N=6	Control	1.0
	BO	1.0
	BO＋Pro	1.0
BO＋Pro N=10	Control	1.0
	BO	1.0
	Pro	1.0

Treatment effects : $P = 0.11$, total sample size = 8
Time effects : $P = 4.3 \times 10^{-14}$, total sample size = 8
Two way ANOVA with repeated measures followed by Bonferroni *post hoc* test

C

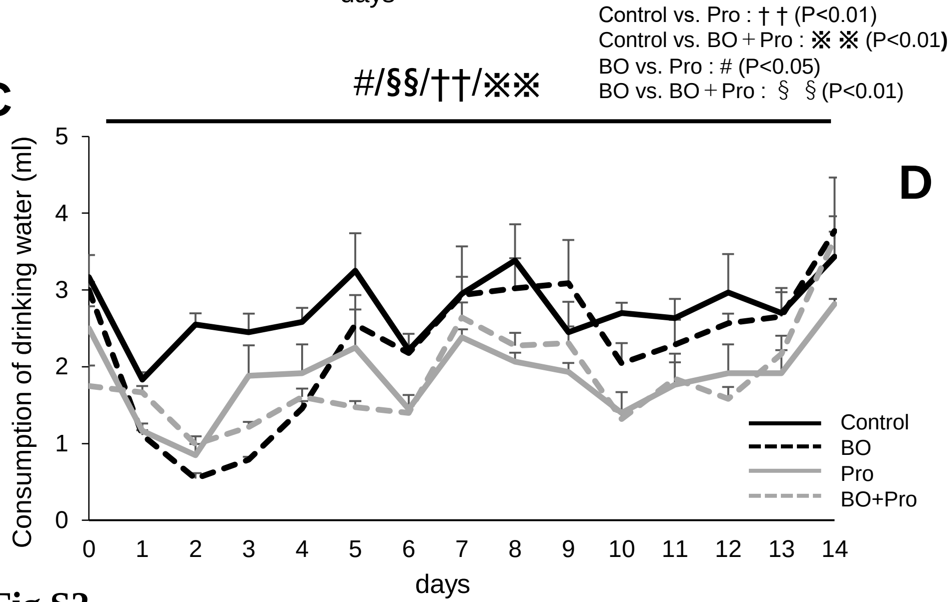

D

		P value (Bonferroni)
Control N=6	BO	6.7×10^{-2}
	Pro	1.5×10^{-5}
	BO＋Pro	2.8×10^{-7}
BO N=8	Control	6.7×10^{-2}
	Pro	1.3×10^{-2}
	BO＋Pro	6.7×10^{-4}
Pro N=6	Control	1.5×10^{-5}
	BO	1.3×10^{-2}
	BO＋Pro	1.0
BO＋Pro N=10	Control	2.8×10^{-7}
	BO	6.7×10^{-4}
	Pro	1.0

Treatment effects : $P = 2.7 \times 10^{-8}$, total sample size = 8
Time effects : $P = 3.3 \times 10^{-10}$, total sample size = 8
Two way ANOVA with repeated measures followed by Bonferroni *post hoc* test

Fig S2.
(A-B) Food consumption **(A)** and its statistical analysis **(B)** throughout 2-week experimental period.
(C-D) Drinking water **(C)** and its statistical analysis **(D)** throughout the 2-week experimental period.

Fig S3

A

		P value (Student T)
Control (N=5)	BO (N=5)	7.6×10^{-4}

Total sample size = 6
Student T test

B

		P value (Bonferroni)
BO − 1day N=5	BO + 1day	1.1×10^{-2}
	BO + 7day	2.3×10^{-3}
	BO + 14day	3.6×10^{-5}
BO + 1day N=5	BO − 1day	1.1×10^{-2}
	BO + 7day	0.6
	BO + 14day	1.0
BO + 7day N=5	BO − 1day	2.3×10^{-3}
	BO + 1day	0.6
	BO + 14day	1.0
BO + 14day N=5	BO − 1day	3.6×10^{-5}
	BO + 1day	1.0
	BO + 7day	1.0

Treatment effects : $P = 2.1 \times 10^{-6}$, total sample size = 8
One way ANOVA with repeated measures followed by Bonferroni *post hoc* test

C

		P value (Bonferroni)
BO − 1day N=5	BO + 1day	5.7×10^{-3}
	BO + 7day	3.0×10^{-3}
	BO + 14day	1.1×10^{-3}
BO + 1day N=5	BO − 1day	5.7×10^{-3}
	BO + 7day	0.9
	BO + 14day	1.0
BO + 7day N=5	BO − 1day	3.0×10^{-3}
	BO + 1day	0.9
	BO + 14day	1.0
BO + 14day N=5	BO − 1day	1.1×10^{-3}
	BO + 1day	1.0
	BO + 7day	1.0

Treatment effects : $P = 1.6 \times 10^{-7}$, total sample size = 8
One way ANOVA with repeated measures followed by Bonferroni *post hoc* test

D

		P value (Bonferroni)
BO − 1day N=5	BO + 1day	3.2×10^{-4}
	BO + 7day	3.6×10^{-5}
	BO + 14day	3.6×10^{-2}
BO + 1day N=5	BO − 1day	3.2×10^{-4}
	BO + 7day	1.0
	BO + 14day	0.1
BO + 7day N=5	BO − 1day	3.6×10^{-5}
	BO + 1day	1.0
	BO + 14day	0.6
BO + 14day N=5	BO − 1day	3.6×10^{-2}
	BO + 1day	0.1
	BO + 7day	0.6

Treatment effects : $P = 6.7 \times 10^{-7}$, total sample size = 8
One way ANOVA with repeated measures followed by Bonferroni *post hoc* test

Fig S3.
(A) Statistical analysis of **Fig 2B**.
(B) Statistical analysis of **Fig 2C**.
(C) Statistical analysis of **Fig 2D**.
(D) Statistical analysis of **Fig 2E**.

Fig S4

A

		P value (Bonferroni)
BO－1day N=5	BO＋1day	2.8×10^{-3}
	BO＋7day	3.3×10^{-2}
	BO＋14day	3.2×10^{-2}
BO＋1day N=5	BO－1day	2.8×10^{-3}
	BO＋7day	1.0
	BO＋14day	5.5×10^{-2}
BO＋7day N=5	BO－1day	3.3×10^{-2}
	BO＋1day	1.0
	BO＋14day	1.0
BO＋14day N=5	BO－1day	3.2×10^{-2}
	BO＋1day	5.5×10^{-2}
	BO＋7day	1.0

Treatment effects : $P = 5.0 \times 10^{-6}$, total sample size = 8
One way ANOVA with repeated measures followed by Bonferroni *post hoc* test

B

		P value (Bonferroni)
BO－1day N=5	BO＋1day	1.6×10^{-56}
	BO＋7day	1.2×10^{-44}
	BO＋14day	7.1×10^{-19}
BO＋1day N=5	BO－1day	1.6×10^{-56}
	BO＋7day	1.2×10^{-4}
	BO＋14day	1.5×10^{-27}
BO＋7day N=5	BO－1day	1.2×10^{-44}
	BO＋1day	1.2×10^{-4}
	BO＋14day	7.2×10^{-15}
BO＋14day N=5	BO－1day	7.1×10^{-19}
	BO＋1day	1.5×10^{-27}
	BO＋7day	7.2×10^{-15}

Treatment effects : $P = 4.5 \times 10^{-60}$, total sample size = 8
Time effects : $P = 0.11$, total sample size = 8
Two way ANOVA with repeated measures followed by Bonferroni *post hoc* test

Fig S4.
(A) Statistical analysis of **Fig 2F**.
(B) Statistical analysis of **Fig 2G**.

Fig S5

A

		P value (Tukey)
Control N=6	BO	1.2×10^{-2}
	Pro	1.0
	BO + Pro	1.0
BO N=6	Control	1.2×10^{-2}
	Pro	5.1×10^{-3}
	BO + Pro	1.5×10^{-2}
Pro N=6	Control	1.0
	BO	5.1×10^{-3}
	BO + Pro	1.0
BO + Pro N=6	Control	1.0
	BO	1.5×10^{-2}
	Pro	1.0

total sample size = 8
One way ANOVA followed by Tukey-Kramer *post hoc* test

B

		P value (Tukey)
Control N=6	BO	7.9×10^{-4}
	Pro	1.0
	BO + Pro	1.0
BO N=6	Control	7.9×10^{-4}
	Pro	4.9×10^{-4}
	BO + Pro	1.1×10^{-3}
Pro N=6	Control	1.0
	BO	4.9×10^{-4}
	BO + Pro	1.0
BO + Pro N=6	Control	1.0
	BO	1.1×10^{-3}
	Pro	1.0

total sample size = 12
One way ANOVA followed by Tukey-Kramer *post hoc* test

Fig S5.
(A) Statistical analysis of **Fig 3B**.
(B) Statistical analysis of **Fig 3D**.

Fig S6

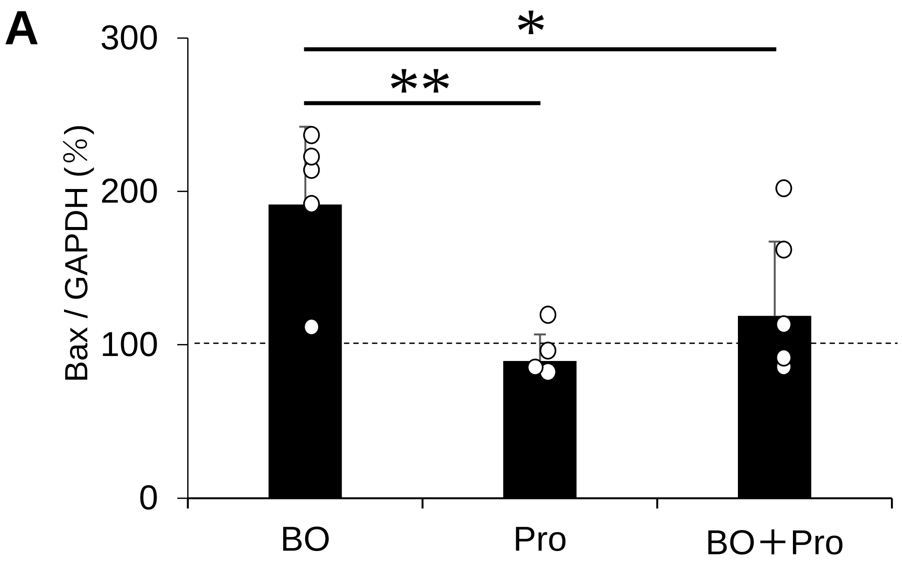

		P value (Tukey)
Control N=4	BO	1.6×10^{-2}
	Pro	1.0
	BO + Pro	0.9
BO N=5	Control	1.6×10^{-2}
	Pro	7.3×10^{-3}
	BO + Pro	3.6×10^{-2}
Pro N=4	Control	1.0
	BO	7.3×10^{-3}
	BO + Pro	0.7
BO + Pro N=6	Control	0.9
	BO	3.6×10^{-2}
	Pro	0.7

total sample size = 8
One way ANOVA followed by Tukey-Kramer *post hoc* test

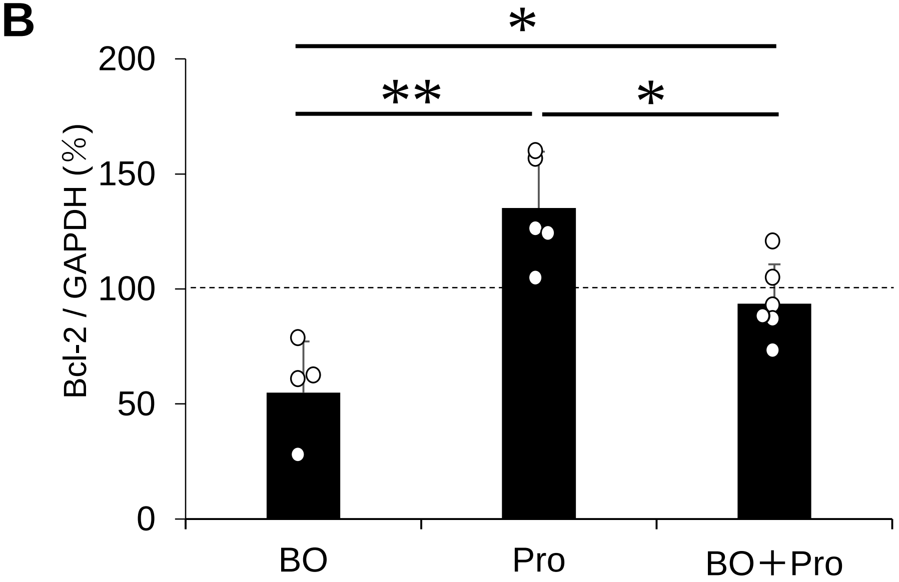

		P value (Tukey)
Control N=4	BO	3.5×10^{-2}
	Pro	0.1
	BO + Pro	1.0
BO N=4	Control	3.5×10^{-2}
	Pro	2.0×10^{-4}
	BO + Pro	5.0×10^{-2}
Pro N=5	Control	0.1
	BO	2.0×10^{-4}
	BO + Pro	2.2×10^{-2}
BO + Pro N=6	Control	1.0
	BO	5.0×10^{-2}
	Pro	2.2×10^{-2}

total sample size = 8
One way ANOVA followed by Tukey-Kramer *post hoc* test

Fig S6.
(A) Expression of Bax (*left*) and its statistical analysis (*right*).
(B) Expression of Bcl-2 (*left*) and its statistical analysis (*right*).

Fig S7

A

		P value (Tukey)
Control N=6	BO	2.4×10^{-3}
	Pro	1.0
	BO + Pro	0.7
BO N=6	Control	2.4×10^{-3}
	Pro	3.5×10^{-3}
	BO + Pro	2.2×10^{-2}
Pro N=5	Control	1.0
	BO	3.5×10^{-3}
	BO + Pro	0.8
BO + Pro N=6	Control	0.7
	BO	2.2×10^{-2}
	Pro	0.8

total sample size = 8
One way ANOVA followed by Tukey-Kramer *post hoc* test

B

		P value (Tukey)
Control N=6	BO	1.0×10^{-2}
	Pro	6.9×10^{-2}
	BO + Pro	1.0
BO N=4	Control	1.0×10^{-2}
	Pro	0.6
	BO + Pro	5.1×10^{-3}
Pro N=6	Control	6.9×10^{-2}
	BO	0.6
	BO + Pro	3.3×10^{-2}
BO + Pro N=6	Control	1.0
	BO	5.1×10^{-3}
	Pro	3.3×10^{-2}

total sample size = 8
One way ANOVA followed by Tukey-Kramer *post hoc* test

C

		P value (Tukey)
Control N=6	BO	9.8×10^{-5}
	Pro	0.8
	BO + Pro	5.2×10^{-2}
BO N=6	Control	9.8×10^{-5}
	Pro	6.5×10^{-5}
	BO + Pro	3.8×10^{-2}
Pro N=4	Control	0.8
	BO	6.5×10^{-5}
	BO + Pro	1.8×10^{-2}
BO + Pro N=6	Control	5.2×10^{-2}
	BO	3.8×10^{-2}
	Pro	1.8×10^{-2}

total sample size = 8
One way ANOVA followed by Tukey-Kramer *post hoc* test

Fig S7.
(A) Statistical analysis of **Fig 4A**. **(B)** Statistical analysis of **Fig 4B**.
(C) Statistical analysis of **Fig 4C**.

Fig S8

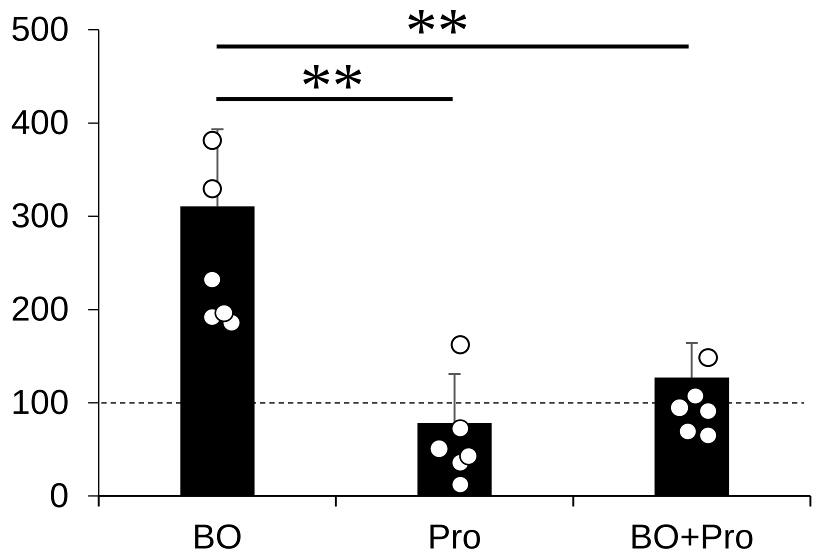

		P value (Tukey)
Control N=6	BO	8.9×10^{-5}
	Pro	0.9
	BO＋Pro	0.9
BO N=6	Control	8.9×10^{-5}
	Pro	2.5×10^{-5}
	BO＋Pro	4.6×10^{-4}
Pro N=6	Control	0.9
	BO	2.5×10^{-5}
	BO＋Pro	0.6
BO＋Pro N=6	Control	0.9
	BO	4.6×10^{-4}
	Pro	0.6

total sample size = 8
One way ANOVA followed by Tukey-Kramer *post hoc* test

Fig S8.
CaMKII phosphorylation (Thr-286) (*left*) and its statistical analysis (*right*).

Fig S9

A

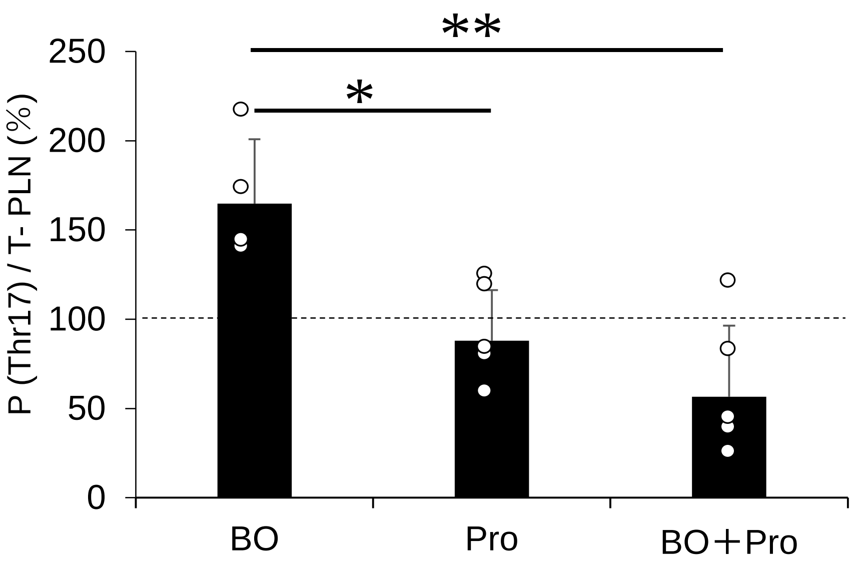

		P value (Tukey)
Control N=5	BO	3.2×10^{-2}
	Pro	0.9
	BO＋Pro	0.2
BO N=4	Control	3.2×10^{-2}
	Pro	1.1×10^{-2}
	BO＋Pro	5.6×10^{-4}
Pro N=5	Control	0.9
	BO	1.1×10^{-2}
	BO＋Pro	0.4
BO＋Pro N=5	Control	0.2
	BO	5.6×10^{-4}
	Pro	0.4

total sample size = 8

One way ANOVA followed by Tukey-Kramer *post hoc* test

B

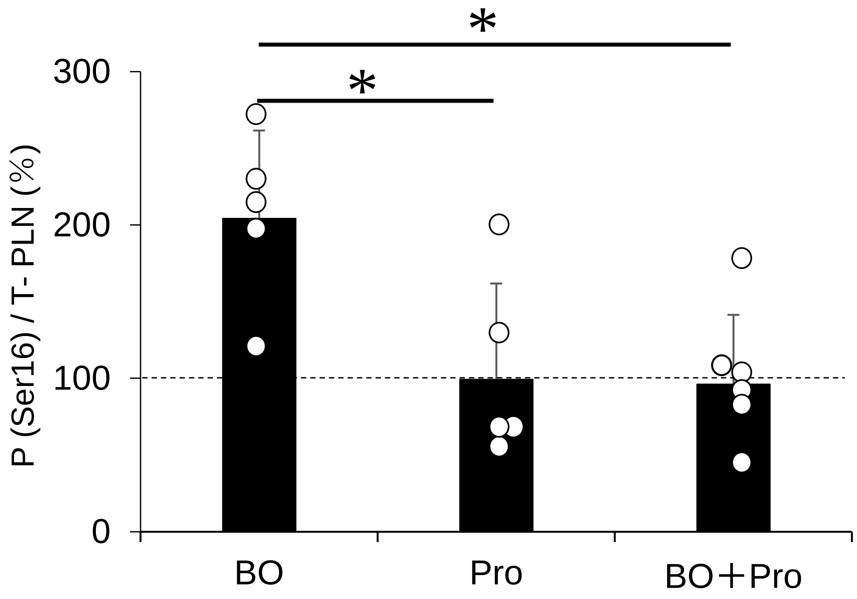

		P value (Tukey)
Control N=5	BO	2.1×10^{-2}
	Pro	1.0
	BO＋Pro	1.0
BO N=5	Control	2.1×10^{-2}
	Pro	2.1×10^{-2}
	BO＋Pro	1.2×10^{-2}
Pro N=5	Control	1.0
	BO	2.1×10^{-2}
	BO＋Pro	1.0
BO＋Pro N=6	Control	1.0
	BO	1.2×10^{-2}
	Pro	1.0

total sample size = 8

One way ANOVA followed by Tukey-Kramer *post hoc* test

Fig S9.

(A) PLN phosphorylation (Thr-17) (*left*) and its statistical analysis (*right*).

(B) PLN phosphorylation (Ser-16) (*left*) and its statistical analysis (*right*).

Fig S10

A

		P value (Tukey)
Control N=6	BO	2.6×10^{-2}
	Pro	1.0
	BO + Pro	1.0
BO N=6	Control	2.6×10^{-2}
	Pro	2.3×10^{-2}
	BO + Pro	2.8×10^{-2}
Pro N=5	Control	1.0
	BO	2.3×10^{-2}
	BO + Pro	1.0
BO + Pro N=6	Control	1.0
	BO	2.8×10^{-2}
	Pro	1.0

total sample size = 8

One way ANOVA followed by Tukey-Kramer *post hoc* test

B

		P value (Tukey)
Control N=6	BO	1.8×10^{-3}
	Pro	1.0
	BO + Pro	0.9
BO N=6	Control	1.8×10^{-3}
	Pro	1.5×10^{-3}
	BO + Pro	1.0×10^{-2}
Pro N=6	Control	1.0
	BO	1.5×10^{-3}
	BO + Pro	0.8
BO + Pro N=6	Control	0.9
	BO	1.0×10^{-2}
	Pro	0.8

total sample size = 8

One way ANOVA followed by Tukey-Kramer *post hoc* test

C

		P value (Tukey)
Control N=6	BO	3.7×10^{-6}
	Pro	0.9
	BO + Pro	0.3
BO N=6	Control	3.7×10^{-6}
	Pro	3.7×10^{-6}
	BO + Pro	1.2×10^{-4}
Pro N=4	Control	0.9
	BO	3.7×10^{-6}
	BO + Pro	0.1
BO + Pro N=6	Control	0.3
	BO	1.2×10^{-4}
	Pro	0.1

total sample size = 8

One way ANOVA followed by Tukey-Kramer *post hoc* test

Fig S10.
(A) Statistical analysis of **Fig 5A**.
(B) Statistical analysis of **Fig 5B**.
(C) Statistical analysis of **Fig 5C**.

Fig S11

A

		P value (Tukey)
Control N=6	BO	3.5×10^{-11}
	Pro	0.6
	BO＋Pro	1.0
BO N=6	Control	3.5×10^{-11}
	Pro	7.8×10^{-12}
	BO＋Pro	5.2×10^{-11}
Pro N=6	Control	0.6
	BO	7.8×10^{-12}
	BO＋Pro	0.4
BO＋Pro N=6	Control	1.0
	BO	5.2×10^{-11}
	Pro	0.4

total sample size = 8
One way ANOVA followed by Tukey-Kramer *post hoc* test

B

		P value (Tukey)
Control N=6	BO	3.8×10^{-3}
	Pro	1.0
	BO＋Pro	1.0
BO N=6	Control	3.8×10^{-3}
	Pro	1.3×10^{-3}
	BO＋Pro	3.4×10^{-3}
Pro N=6	Control	1.0
	BO	1.3×10^{-3}
	BO＋Pro	1.0
BO＋Pro N=6	Control	1.0
	BO	3.4×10^{-3}
	Pro	1.0

total sample size = 16
One way ANOVA followed by Tukey-Kramer *post hoc* test

C

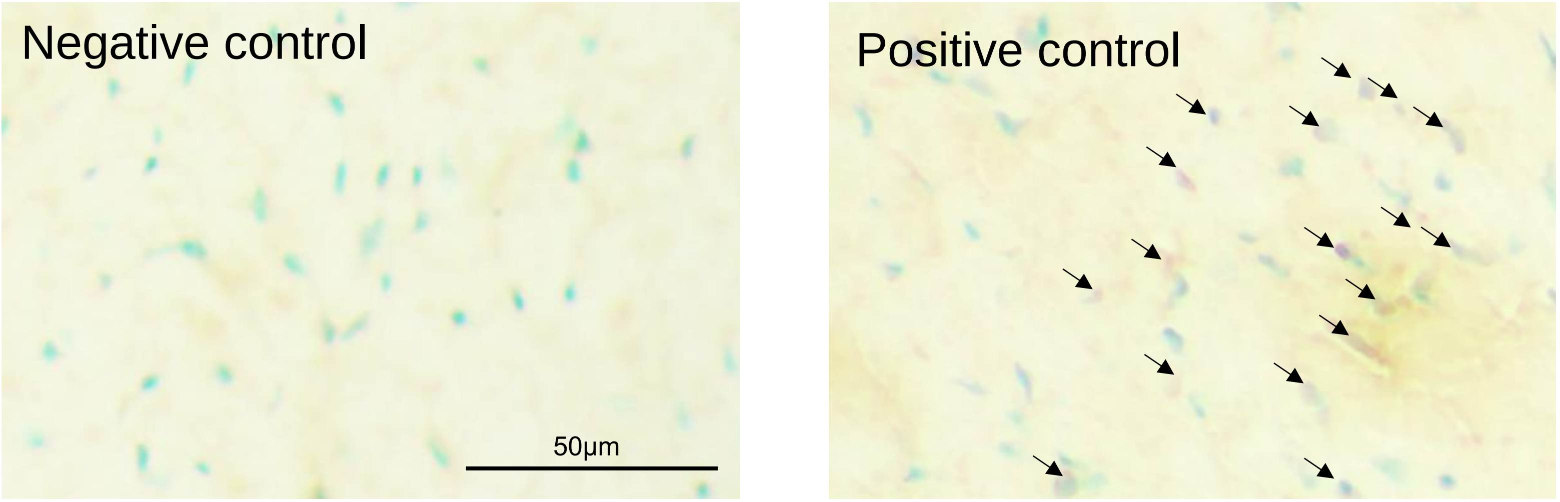

Fig S9.
(A) Statistical analysis of **Fig 6B**. **(B)** Statistical analysis of **Fig 6D**.
(C) Representative images of negative (left) and positive (right) controls of 8-OHdG immunostaining.

Fig S12

A

		P value (Tukey)
Control N=6	BO	2.5×10^{-2}
	Pro	1.0
	BO＋Pro	1.9×10^{-3}
BO N=8	Control	2.5×10^{-2}
	Pro	3.7×10^{-2}
	BO＋Pro	0.8
Pro N=6	Control	1.0
	BO	3.7×10^{-2}
	BO＋Pro	3.1×10^{-3}
BO＋Pro N=10	Control	1.9×10^{-3}
	BO	0.8
	Pro	3.1×10^{-3}

total sample size = 16
One way ANOVA followed by Tukey-Kramer *post hoc* test

B

		P value (Tukey)
Control N=6	BO	0.5
	Pro	1.0
	BO＋Pro	0.7
BO N=8	Control	0.5
	Pro	0.3
	BO＋Pro	0.9
Pro N=6	Control	1.0
	BO	0.3
	BO＋Pro	0.5
BO＋Pro N=10	Control	0.7
	BO	0.9
	Pro	0.5

total sample size = 72
One way ANOVA followed by Tukey-Kramer *post hoc* test

C

		P value (Tukey)
Control N=6	BO	0.5
	Pro	1.0
	BO＋Pro	0.7
BO N=8	Control	0.5
	Pro	0.4
	BO＋Pro	1.0
Pro N=6	Control	1.0
	BO	0.4
	BO＋Pro	0.6
BO＋Pro N=10	Control	0.7
	BO	1.0
	Pro	0.6

total sample size = 80
One way ANOVA followed by Tukey-Kramer *post hoc* test

D

		P value (Tukey)
Control N=6	BO	1.0
	Pro	1.0
	BO＋Pro	0.9
BO N=6	Control	1.0
	Pro	0.8
	BO＋Pro	0.7
Pro N=4	Control	1.0
	BO	0.8
	BO＋Pro	1.0
BO＋Pro N=6	Control	0.9
	BO	0.7
	Pro	1.0

total sample size = 196
One way ANOVA followed by Tukey-Kramer *post hoc* test

Fig S12.
(A) Statistical analysis of **Table1 Body weight**. **(B)** Statistical analysis of **Table1 CM weight**.
(C) Statistical analysis of **Table1 CM weight/tibia length**. **(D)** Statistical analysis of **Table1 CM weight/body weight**.

Fig S13

A

		P value (Tukey)
Control N=10	BO	7.1×10^{-2}
	Pro	0.2
	BO＋Pro	1.0
BO N=7	Control	7.1×10^{-2}
	Pro	1.0
	BO＋Pro	9.5×10^{-2}
Pro N=5	Control	0.2
	BO	1.0
	BO＋Pro	0.3
BO＋Pro N=7	Control	1.0
	BO	9.5×10^{-2}
	Pro	0.3

total sample size = 32
One way ANOVA followed by Tukey-Kramer *post hoc* test

B

		P value (Tukey)
Control N=10	BO	1.0
	Pro	0.5
	BO＋Pro	0.8
BO N=7	Control	1.0
	Pro	0.7
	BO＋Pro	1.0
Pro N=5	Control	0.5
	BO	0.7
	BO＋Pro	0.9
BO＋Pro N=7	Control	0.8
	BO	1.0
	Pro	0.9

total sample size = 120
One way ANOVA followed by Tukey-Kramer *post hoc* test

C

		P value (Tukey)
Control N=10	BO	1.1×10^{-7}
	Pro	3.1×10^{-9}
	BO＋Pro	0.3
BO N=7	Control	1.1×10^{-7}
	Pro	0.1
	BO＋Pro	3.1×10^{-5}
Pro N=5	Control	3.1×10^{-9}
	BO	0.1
	BO＋Pro	4.0×10^{-7}
BO＋Pro N=7	Control	0.3
	BO	3.1×10^{-5}
	Pro	4.0×10^{-7}

total sample size = 8
One way ANOVA followed by Tukey-Kramer *post hoc* test

D

		P value (Tukey)
Control N=10	BO	2.7×10^{-7}
	Pro	1.2×10^{-8}
	BO＋Pro	0.2
BO N=7	Control	2.7×10^{-7}
	Pro	0.2
	BO＋Pro	9.1×10^{-5}
Pro N=5	Control	1.2×10^{-8}
	BO	0.2
	BO＋Pro	1.8×10^{-6}
BO＋Pro N=7	Control	0.2
	BO	9.1×10^{-5}
	Pro	1.8×10^{-6}

total sample size = 8
One way ANOVA followed by Tukey-Kramer *post hoc* test

Fig S13.
(A) Statistical analysis of **Table1 LVEDD**. **(B)** Statistical analysis of **Table1 LVESD**.
(C) Statistical analysis of **Table1 LVEF**. **(D)** Statistical analysis of **Table1 %FS**.
