# Supplementary material for: Effects of occlusal disharmony on cardiac fibrosis, myocyte apoptosis and myocyte oxidative DNA damage in mice
Source: PLoS One. 2020 Jul 27;15(7):e0236547. doi: 10.1371/journal.pone.0236547 (PMC7384634; doi:10.1371/journal.pone.0236547)
Supplement: Attachment — Submitted filename: Response 2nd revised PLOS ONE-01-add RS correction-02.docx [file pone.0236547.s004.docx]

***Reviewer #2: The paper was overall improved. However, there are some issues. The paper should be revised.***

TITLE

The papers used homeostasis, but it is not appropriate and not concrete. For example, “Effects of occlusal disharmony on cardiac fibrosis, myocyte apoptosis and myocyte oxidative DNA damage in mice”.

Response:

We modified the title as the reviewer suggested.

***Please add the timing of serum corticosterone measurements and/or collection. The collection should be in the morning.***

Response:

We added the following sentences in the revised manuscript with a new reference (Page 8, Lines 9-10).

Blood sampling was done in the morning (9:00-10:00AM) and the procedure was completed within 30 s from the time of contact with the mouse [1].

***Editor: --- I strongly recommend the authors to ----disclose the use of previously published data in the manuscript---, or significantly change the manuscript with great care for handling raw data, at least without showing the membrane and images, ---***

Response:

We sincerely apologize for omitting to mention our previous paper written in Japanese (Circ Cont 2020). Although we had described the BO-promoted increase of Bax/Bcl-2 ratio, CaMKII phosphorylation (Thr-286) and PLN phosphorylation (Ser-16, Thr-17) in that paper (Circ Cont 2020), it did not describe the effects of propranolol on the BO-promoted increase of these molecules, which are critical for the present manuscript. Accordingly, we adopted your second suggestion to avoid duplication, and have modified **Fig 4A**, **Fig 4B**, **Fig 4D**, **Fig 5A** and **Fig 5B** in the original version and incorporated the modified figures in the revised manuscript as **Fig S6**, **Fig S8** and **Fig S9**. Thus, the revised manuscript no longer duplicates material from the previous paper. We apologize for having failed to spot this before.

We also modified parts in the results section as shown below.

1) Page 23, Lines 10-16

Expression of Bax, an accelerator of apoptosis, in the heart was significantly increased by BO treatment --- in accordance with the previous study **(Fig S6A)** [2]. Propranolol alone had no effect on Bax expression, but blocked the BO-induced increase--- **(Fig S6A)**.

2) Page 23, Line 17-Page 24, Line 5

We also found that the expression of Bcl-2, a decelerator of apoptosis, in cardiac muscle was significantly decreased by BO treatment (Control (*n* = 4) vs. BO (*n* = 4); 100 ± 19 vs. 55 ± 22 %, *P* = 3.5 x 10^-2^ by one-way ANOVA followed by the Tukey-Kramer *post hoc* test) in accordance with the previous study **(Fig S6B)** [2]. Propranolol alone had no effect on the Bcl-2 expression, but blocked the BO-induced decrease --- **(Fig S6B)**.

3) Page 25, Lines 6-12

We thus examined the amounts of phospho-CaMKII (Thr-286) in the heart of BO mice and found that it was significantly increased --- in accordance with the previous study **(Fig S8)** [2]. Propranolol alone had no effect on the amounts of phospho-CaMKII (Thr-286), but propranolol blocked this increase --- **(Fig S8)**.

4) Page 26, Lines 3-12

Phospho-PLN (Thr-17) and phospho-PLN (Ser-16) were significantly increased in cardiac muscle of BO mice--- in accordance with the previous study **(Fig S9A and S9B)** [2]. Propranolol alone had no effect on the amounts of phospho-PLN (Thr-17 and Ser-16), but propranolol blocked both phosphorylations--- **(Fig S9A and S9B)**.

**References**

1. Grootendorst J, Oitzl MS, Dalm S, Enthoven L, Schachner M, et al. (2001) Stress alleviates reduced expression of cell adhesion molecules (NCAM, L1), and deficits in learning and corticosterone regulation of apolipoprotein E knockout mice. Eur J Neurosci 14: 1505-1514.

2. Yagisawa Y, Suita K, Ohnuki Y, Ito A, Umeki D, et al. (2020) Effects of experimental malocclusion on cardiac function inmice. Circ Cont 41: 38-45.
